# The Herbst Clinics in America

**Published:** 1886-09

**Authors:** 


### THE HERBST CLINICS IN AMERICA.

REPORTED EXPRESSLY FOR THE INDEPENDENT PRACTITIONER.

Clinic given on Saturday, July 3d, at the office of Dr. Bödecker.

Dr. Herbst filled with gold cylinders, for Dr. Bödecker, a labial cavity in the right upper lateral incisor. It being exceedingly sensitive, an obtundent, original with Dr. Herbst, was applied. This is made in the following manner: A quantity of chemically pure sulphuric acid is put into a small test tube and saturated with cocaine. Then, while the mixture is stirred by means of a glass rod, sulphuric ether is poured in to the point of super-saturation; if any ether remains on the surface it will evaporate by itself, or it may be expelled by means of a chip-blower. A drop of this obtundent, upon a piece of cotton, was applied to the sensitive labial cavity with great success, the cavity being excavated without pain. After the preparation of the cavity, it was cleaned, disinfected as usual, and filled with Wolrab's gold, in four minutes and a half.

Dr. J. C. Sproul then took the operating chair, and Dr. Herbst filled for him the right upper second bicuspid, the cavity occupying the distal and grinding surface of the tooth. Previous to the excavation a matrix of German silver was prepared in the following manner: A piece of German silver, No. 32 American gauge, onequarter of an inch in width and one and one-half inches in length, was passed around the bicuspid to be filled. Then, by means of a blunt pair of cutting forceps, which in form somewhat resemble a pair of flat-nosed pliers, it was drawn around the tooth in such a way as to make it fit very closely. The German silver ring was then withdrawn from the tooth, a little soldering fluid (solution of chloride of zinc) was applied to the flanges and the ring soldered. One of the principal points to be remembered in the making of this matrix is to avoid allowing the tin solder to run to the side of the matrix next the cavity to be filled, as when the tin is touched by the rotating instrument some of it will be incorporated into the gold and the cohesion of the separate layers of gold impaired. Previous to soldering, the matrix must be made perfectly clean, and this is done by means of a piece of cotton wound around an engine bur dipped into moistened pumice stone, and this rubbed over the ring, inside as well as outside. After the cavity had been thoroughly excavated, the rubber dam and the matrix were applied and the filling introduced upon the cervical wall of the cavity. Dr. Herbst placed a very thin layer of tin, burnishing it up over the pulp, which was very nearly exposed, thereby modifying thermal changes. The tin was then followed by a layer of Wolrab's gold cylinders, No. 0, condensed in the usual way, first with the hand instrument and then with an agate point in the dental engine. When this was completed, a fine instrument made of a broken excavator was pressed all over the surface, first to discover all imperfectly condensed places, and secondly to roughen the polished surfaces of the gold, thereby obtaining better unity of the layers of the filling. The introduction of the gold occupied about eighteen minutes. The gentlemen present at this meeting were Drs. Taft, Bonwill, Abbott, McKellops, Rehwinkel, Tennison, Andrews, V. Pressler and Rhein.

In the afternoon another clinic was given, at which Dr. Herbst filled a lower molar handed to him by Dr. Dwinelle, which had been extracted over thirteen years, the cavity occupying the mesial and grinding surface of the tooth. Previous to excavating the cavity, a German silver matrix was applied, as mentioned before, but in addition to the matrix a brass wire was soldered all around it with soft solder, in order to stiffen it. After the cavity had been excavated, the tooth was imbedded, with the matrix in position, in a large piece of shellac. The first layers of gold were condensed by means of a piece of cotton in the following manner: Cotton enough to fill the cavity was put over the gold, and then, by means of a large smooth engine burnisher, it was burnished into every corner where there was any gold; when the cotton was removed, it showed that the gold was thoroughly depressed into every portion of the cavity, and it was then further condensed by means of agate instruments in the dental engine. Dr. Herbst here remarked that the cotton, when applied in this manner, will make the most cohesive gold non-cohesive, but the moment rotating instruments have been used upon the surface it will again become as cohesive as before. The rest of the layers of gold were applied in the usual way, until the cavity was very nearly full, when one of the walls of the tooth gave way on account of the desiccated condition of the dry tooth, and the defect had to be repaired with gold, without removing the matrix, although the edges of the tooth could not be made perfectly smooth. When it was filled, the shellac and the matrix were removed, and the gold was found to be absolutely perfect in every respect. The filling was then finished with sandpaper disks, without burnishing the outer surface, so as to see whether the gold had reached all the rough edges of the broken wall. It was very critically examined by Drs. Taft, Dwinelle, Rehwinkel, Parr, Northrop and Bogue, all of whom agreed that the operation was absolutely perfect.

Dr. Herbst then filled a cavity in a steel matrix (the same one used by Dr. E. Parmley Brown for ascertaining the comparative weight of gold when condensed by the different methods of filling teeth), into which he introduced Wolrab's gold cylinders, No. 0, by means of hand instruments, assisted by his new agate points. This matrix was filled in about nineteen minutes, and the filling, when completed, weighed eighteen and one-sixteenth grains, or, one-sixteenth of a grain more than the filling introduced by Dr. Brown, with the electric mallet and an extra powerful battery, in forty minutes time.

Dr. Dwinelle, to satisfy himself as to the possibility of condensing gold by means of a piece of cotton, took an engraved stone, and after he had introduced a few large Wolrab cylinders, applied a piece of cotton over it and condensed the gold with it. When removed from the matrix, the gold was found to be a beautiful and exact counterpart of the engraved stone.

CLINIC GIVEN MONDAY, JULY 5TH, AT THE OFFICE OF DR. C. F. W. BÖDECKER.

Dr. Herbst filled for Miss Tennison a very large cavity in the left lower first molar, occupying the mesial distal and grinding surface. The tooth had been devitalized, and its pulp canals filled by Dr. Tennison some time previous. Before the temporary filling was removed a German silver matrix was applied, which, as there was plenty of space between the first molar and second bicuspid, was strengthened by means of tin solder, when the cavity was prepared in the usual manner, the rubber dam applied and the matrix readjusted, the filling of this cavity occupying forty-five minutes. At the cervical wall of the distal surface was placed a thin layer of tin, which was followed by large Wolrab gold cylinders. The gentlemen who witnessed this operation were Drs. Atkinson, Bogue, Dwinelle, Tennison, Mills, Rhein, Rehwinkel, Taft, Meiggs, Sichel and V. Pressler, all of whom expressed great satisfaction at the result of the operation.

CLINIC GIVEN JULY 6TH AT THE NEW YORK COLLEGE OF DENTISTRY, CORNER OF 2D AVENUE AND 23D STREET, FROM 12.30 TILL 2.30 P. M.

Dr. Herbst first explained the general principles of filling by rotating instruments in the engine, as follows: All the gold introduced into the first layer of a cavity should be compressed in such a manner that it will not roll up on its sides, which may be prevented by using a large quantity of gold and a very large hand instrument, as large as the cavity will admit. If the cavity is shallow and very large, it is best to resort to the condensation by means of a piece of cotton, as before mentioned. After the first layer has been put into the cavity in this manner, it should be condensed with a fine instrument in the dental engine, made either of agate, blood-stone, garnet or steel. Steel instruments, however, when the engine is run at high speed, will become coated with a thin film of gold and thereby roughened, and while rotating will, by friction, heat the filling to such a degree as to make it uncomfortable, and at times even painful. In small cavities, such as incisor teeth, stone instruments will be found too large, and steel is the only material employed. If a steel instrument becomes coated with gold, it should be rubbed upon a piece of No. 1 sandpaper frequently, when friction will be prevented.

Dr. Herbst then filled with gold, before the students of the college, a steel matrix made of four parts. When completed the adaptation was found to be absolutely perfect. He then exhibited to the students what may be called his museum. Eighteen gentlemen were in attendance.

CLINIC GIVEN AT THE DEPOT OF S. S. WHITE DENTAL MANUFACTURING COMPANY, CORNER OF 9TH STREET AND BROADWAY, JULY 6TH, AT 3 P. M., UNDER THE AUSPICES OF THE FIRST DISTRICT DENTAL SOCIETY OF THE STATE OF NEW YORK.

Dr. Herbst filled for Dr. Bödecker a left upper central incisor. The cavity was found to be very shallow, occupying the labial portion of the tooth. As it was exceedingly sensitive, Dr. Herbst's obtundent was applied, and the tooth was excavated with but very slight pain to the patient. After the rubber dam had been applied a clamp was used to hold the rubber above the cavity in the cervical portion of the tooth, which was original with the operator. It was made from a curtain ring, to one side of which was soldered a foot-like process, while a flange was soldered to the other. Before applying this clamp, Dr. Herbst inserted two pieces of German silver, one between the cutting edges of the two central incisors, extending downward about a quarter of an inch, which was twisted half way around. The other was inserted between the eye-tooth and lateral in the same manner, when some impression material, which ought to be of the hard grade, was softened and pressed over the lingual and cutting edges of the tooth. When hard, the clamp was heated and pressed into position above the cervical margin of the cavity in the central incisor tooth, carrying the rubber dam before it. The clamp being put into position, the cavity was filled in about twenty-three minutes. After the tooth had been filled, his appliances and specimens of filled teeth were exhibited. This display comprised of five trays. In the first were seen all possible cases of cavities illustrated; some of these preparations had been previously exhibited at the clinics and meeting of the First District Dental Society by Dr. Bödecker, and some of them have already been described in the INDEPENDENT PRACTI-

TIONER. The cavities thus illustrated by preparations were grinding surfaces in molars and bicuspids, cavities in the distal, mesial and grinding surface of a bicuspid, illustrating the application of a steel spring matrix combined with shellac, in the following manner:

There was a large space between a bicuspid and molar tooth, which was filled up by shellac pressing against the steel spring matrix, placed between the distal cavity in the second bicuspid and the mesial surface of the second molar tooth. Another cavity in the distal portion of the first bicuspid was also filled up by shellac pressing against another steel spring matrix. Thus the second bicuspid tooth was almost encircled by two pieces of steel spring held very firmly in position by shellac. The mesial part of this matrix, however, did not quite reach the grinding surface of the tooth, as it would obstruct the entrance of the gold in the mesial cavity. On the distal surface the matrix projected a little above the grinding surface of the tooth. This form of matrix should only be employed when the German silver matrix is not applicable.

In another instance three teeth were exhibited with cavities like those already described, but in this case German silver matrices had been applied around the tooth, strengthened with brass wire and tin solder. One of the preparations comprised two upper central incisors, both with cavities involving the mesial and a little of the lingual surfaces, with a matrix prepared of shellac and steel in the following manner: The shellac had been softened over the flame of an alcohol lamp, pressed against the lingual surface and a little over the cutting edges of the two front teeth, when it was removed, and a small piece of steel watch spring inserted between the places of the two cavities in the centrals; the steel spring in this instance did not quite reach the labial border of the cavity, as it would have obstructed the entrance of these cavities. In the next preparation were observed the two above described fillings completed. Another preparation showed the matrix as applied for a contour operation of an upper canine and lateral tooth. The shellac matrix, which must be made previous to excavating the cavity, had been prepared in the method described above, but in addition to this a steel spring was adjusted at the mesial surface of the eyetooth, and another piece of steel spring was adjusted over the cutting edge, thus representing an entirely closed cavity with four parts into which the gold could be easily packed. The next preparation showed the completion of this operation as described before. There were ten other preparations, showing materially the same principles in different localities of teeth. Another preparation showed the application of a steel spring for the use of approximate cavities between incisors and bicuspid teeth. Dr. Herbst distributed a number of these steel springs at every clinic, to every gentleman present, and described their use in the following way: A steel spring about six to ten inches long, with a little piece of wire soldered across the end so as to prevent it from slipping from between the teeth, is inserted next the approximate cavity. This steel spring will serve either as a matrix or as a protection for the proximate surface of the adjoining tooth during excavation. As a protection for the adjoining tooth it is an exceedingly valuable adjunct in cases where there is a filling, or when it contains a cavity which is to be excavated. Even if the tooth be not decayed, it will prevent the marring of it by the bur during this process, though the bur will suffer somewhat. This piece of steel spring is put between the teeth, and bent over the tooth which is to be shielded during the process of excavation or filling. If the necks of the teeth, especially those of lower bicuspids, are very small, the steel spring has a tendency to slip down upon the gum and produce pain, besides being a disadvantage, as it is not perfectly steady in that position. When this is the case, one of the ends of the steel spring is inserted into a small piece of previously softened shellac placed between the teeth, and drawn forward, at the same time pressing the shellac somewhat over the grinding surfaces of the teeth. When cold this will slip into its place and prevent any possible movement of the steel spring matrix, either up or down. In every case where shellac is made use of, the operator ought to be very careful not to let particles of it adhere to the walls of the matrix, as the rotating instruments will incorporate this into the gold and prevent the second layer from cohering to the first. If the gold is packed against the shellac matrix, it ought to cover the entire surface of the shellac in the first layer, thus preventing the rotating instruments from touching the shellac.

In another tray were observed four molars which had been filled by fitting a piece of 24 carat gold, gauge No. 32, to the cavity to be filled. Upon the lower surface of this was soldered a small ring, cut from a former spiral gold spring. After the cavity had been filled with oxy-phosphate, the gold cap was pressed into it and thoroughly burnished against the edges of the cavity, thus sealing the opening of the cavity by means of a gold cap.

In another preparation was exhibited a matrix made out of a piece of steel, about one and a half inches in length, bent together. This was inserted between the two bicuspids; into the open end, and near the gum, was pushed a small piece of wood, to fasten it and thoroughly hold it against the two cavities in the proximate surfaces of these teeth. There were several of these cavities illustrated in the same manner, which do not require further mention. In some preparations exhibited upon this tray, were teeth that had been filled with Herbst's amalgam, which contains ten per cent. of gold. These teeth, after they had been filled, were split. They showed very minutely the beautiful adaptation of this amalgam to the walls of the cavities. There were also three forms made out of steel springs, one of them about an inch square and a sixteenth of an inch high. These forms had been filled with Herbst's amalgam for the purpose of showing that, during the process of setting, it did not shrink a particle from the walls.

In some instances, when it is desired to fill with amalgam front teeth with thin walls, the usual discoloration is obviated by lining the cavity with a thin layer of gold, as follows: Two or three of the largest gold cylinders are closely compressed between the fingers, and, when perfectly flat, are dipped into a very thin solution of gum copal and sulphuric ether (two grains of copal to half an ounce of ether). When nearly dry the gold is held before the cavity by a pair of tweezers, and by means of a piece of cotton and a rotating instrument in the engine it is put in position and burnished closely against the wall of the cavity. Care must be exercised that the cotton does not wind around the rotating instrument, else it may tear the gold out again. The object of applying a thin layer of copal varnish upon the gold is to protect it from the action of mercury. Teeth thus lined and filled with amalgam do not discolor, as the gold is protected from the action of the mercury by the application of the thin copal varnish. There were many preparations which had been made in this manner, in some of which the gold was visible through the enamel. Some of these teeth had been filled a year ago, yet the tooth had preserved its natural color. All of these preparations were kept moist by water. Another preparation, a central incisor, exhibited a filling upon its labial surface nearly three-quarters of an inch in length, and the whole width of the tooth. By the side of this filling there was a tooth formed out of ivory, which was perfectly flat and polished upon its surface, exhibiting a very small groove around the edge. Dr. Herbst declared that the cavity in the previously described tooth had been prepared in the same manner as this one, the first layer of gold having been packed with cotton, as before alluded to. The filling was exceedingly hard and perfect about the edges.

In this tray, beside a great quantity of other fillings, there was some rubber work which had been lined upon its lingual surface with gold. The lining had been accomplished by taking an ordinary piece of red rubber dissolved in chloroform, and applying this to a sheet of ordinary gold foil. After the flask had been packed with the rubber, a piece of very thin rubber dam was washed, and while wet was laid over the rubber, and the two parts of the flask brought together and very nearly closed. The flask was then carefully separated again, the piece of rubber dam removed, and the gold foil, with its prepared side toward the rubber, placed over the lingual surface of the plaster model, when the flask was again closed and vulcanized in the usual way. Dr. Herbst claims that when the rubber during the process of vulcanization does not come in contact with the plaster, the contours of the model are better preserved, and the piece presents a better appearance when finished.

In tray No. 3, mainly polished materials were exhibited, and these have been described in the INDEPENDENT PRACTITIONER. Some of them have, however, since been improved. Instead of using steel mandrels for holding the rubber and corundum points, Dr. Herbst makes use of German silver, which combines better with the rubber and corundum when vulcanized. They are made in the following manner: One part of ordinary rubber and two parts of corundum or emery are taken. The latter is moistened with chloroform and kneaded into the rubber upon a warm marble slab. When all the corundum has been incorporated into the rubber, it is rolled up into strings of the thickness required, placed in a pill machine and divided into pieces about the size of an ordinary pea. These pieces of rubber are then placed upon a mandrel of German silver which has been previously flattened on its point. A little heat is applied to the mandrel, and it is then inserted into the rubber and corundum. To center the piece of rubber upon the mandrel, Dr. Herbst employs an ingot or bullet mould in the form of a pair of pliers. When they have been centered in this manner, they are flasked and vulcanized in the ordinary way. In this tray were also exhibited some new forms of agate and garnet burnishers, all having been made by Dr. Herbst. The agate points were so set that only a little of the agate protruded from the steel setting, thus very much lessening the danger of breakage. The garnets were made of ordinary garnet beads, by cementing an ordinary broken bur into the hole in the bead by means of common sulphur. When a bead has thus been fastened upon a mandrel, it is ground upon a fine corundum stone, and is then ready for use. The garnet, as well as the agate points, should never be polished, as they will give a polished surface to the gold upon which they are used, and prevent another layer from cohering readily.

Tray No. 4 contained a number of inventions. There was an upper jaw demonstrating the method of holding a replanted tooth in position. This is done in the following manner : Take a piece of rubber dam about one inch in length and about half an inch wide, and depress its center by means of a heated instrument with a round head. Two, three or four holes, according to the number of teeth over which the rubber is to be carried, are made in the rubber when it is applied. The depression in the rubber goes over the crown of the tooth to be replanted. Ligatures are passed around the tooth to hold the piece of rubber in proper position, which ought to remain in place from three to four days, when it will be found that the tooth is sufficiently firm to remain in place. One of the principal advantages of this method of replanting is that the tooth will be pressed into the alveolus a little deeper than its neighbors, thus avoiding the danger from undue pressure of the opposite tooth.

Tooth brushes for the dental engine, to be used in cleaning teeth, are made as follows : Take three small bottle-cleaning brushes, which are about two inches in length, twist the wire firmly and divide them into the required length. Shellac one end of each piece into a plain porte polishing mandrel, while at the other the wires are soldered together with tin solder.

As Dr. Herbst is not near a dental depot, and has not always a porte polisher handy, when he wants one he makes it out of a pencil holder by sawing the point off and soldering it upon a common

German silver mandrel. He cements a piece of sponge into the rest of the pencil holder, and uses it for keeping corundum points moist when grinding and polishing gold fillings.

Diamond discs and diamond saws are easily made by powdering some diamond chips in a steel mortar made for that purpose, then mixing the diamond dust with oil and hammering it into a piece of German silver, which may be used either as a saw or wheel. Diamond drills can be very easily made by taking a German silver mandrel and sawing a slot about one-eighth of an inch in length into the end. A small flat piece of diamond is then pushed into the slot, the ends are bent together and it is soldered with tin solder. The diamond drill may then be formed by rubbing it while revolving in the engine upon a piece of sandpaper. This will remove only the German silver, and the diamond will come to the surface; but this drill does not cut dentine or enamel as well as steel instruments. It is intended more for the use of glass and porcelain.

For making a very small metal plate, without preparing zinc casts, Dr. Herbst takes two impressions of the same mouth, and pours one of them with plaster and the other with plaster and pumice stone. He burnishes a piece of platinum foil over the plaster model, and over this foil he burnishes a piece of platinum gauze as large as he desires his plate to be. Removing this plate to the model made of pumice stone and plaster, and pinning it down with platinum pins, the foil and gauze is heated, and eighteen carat gold is flowed over it. Thus a very strong and serviceable gold plate may be made in a very few minutes. Pivot teeth may be prepared in the same way.

Plain teeth may be readily made into gum teeth by melting upon the end a little powdered ruby glass. This glass must be very finely powdered under water in an agate mortar, and when fine enough it is applied by means of a camel's hair brush to the cervical portion of the plain tooth, and put into a steel thimble, which is then placed upon charcoal and the glass flowed by means of the ordinary gas flame and a blow pipe. In the same manner a piece of glass may be inserted into a cavity, as has been done with pieces of porcelain in this country, in the following manner :

An impression of the defective cavity in the tooth is taken with wax, into which plaster is poured. This, when dry, is removed and represents the exact cavity of the defective tooth. Very finely powdered white and brown glass is placed into this plaster model. The proportions of the glass are four or five of the ordinary white arsenious acid glass to one of dark brown. Both of these are powdered separately in water in an agate mortar, and then mixed according to the shade required. Some of this powder when moist is introduced into the cavity in the plaster model of the tooth, and placed in the iron thimble and burned. When cold, a little more is added; it is burned again, and if the color is right it may be completed in the third bake, and the piece of glass may be cemented into the cavity by means of oxy-phosphate. If the joint of the glass with the tooth is objectionable, this may be hidden by drilling a little more of the tooth away, obtaining sufficient undercut and filling the cavity with gold in the usual manner.

Dr. Herbst makes all of his nerve broaches of 14 carat gold wire. He points the ends of a wire and fastens it in a piece of impression material by heating. When cold, he cuts the barbs by means of a very sharp chisel, when they are removed and are ready for use.

If an engine bur or drill breaks and is yet long enough for service, he grinds a triangular point upon it and uses it for drilling out fissures, and finds them exceedingly useful and serviceable.

For filling cervical cavities in front teeth he does not make use of the ordinary Evans clamp, but makes one of a steel pen or a curtain ring. A steel pen is broken, annealed and somewhat flattened out. A hole is cut near its end, which is bent over toward the hollow of the steel pen. This bent piece will hold the rubber above the cavity, while the other end of the pen is fastened into a piece of impression material placed over three or five teeth. To secure this impression material in its place, two pieces of metal are pushed between the cutting edges of the teeth and turned around. This will thus prevent the moving of the impression material. The curtain ring may be used for the same purpose by soldering a little leg on one side and a flange of metal on the other. This little leg should be soldered in such a manner that when the ring lies flat the leg is standing up, while the flange lies parallel with the ring. This clamp, when applied, must be heated and the flange pressed into the impression material.

For regulating teeth when a plate is employed, it will be found that sometimes the patient will not keep the plate in the mouth. Dr. Herbst lets the rubber plate extend over the buccal surfaces of the molar teeth, and inserts two, three or four screws near the cervical edge, between the teeth, through the rubber plate. In this manner the plate is held in position very securely, and the wedging may be done in half the usual time by means of cotton.

For repairing rubber plates quickly, Dr. Herbst drills two holes about the thickness of the ordinary mandrel into the rubber plate, in a direction to correspond to a dove-tail. These drill holes he then fills with a round piece of celluloid, letting it extend somewhat upon the labial surface of the teeth. Then the tooth is warmed, held over an alcohol lamp, and as soon as the celluloid softens upon the outer surface of the tooth, it is pressed into position and held there until it is cold. A tooth replaced in this manner will serve almost as well as though it was repaired by vulcanizing, and it can be set in a few minutes.

There was also a box of preparations sent to this country by Dr. Franz Berggren, of Stockholm, a pupil of Dr. Herbst, containing some beautiful fillings which were made out of the mouth, all by the Herbst method. Preparation No. 1 represents a root of an upper first molar tooth, which had been built up. This filling weighed six and a quarter grams. The gold used was Wolrab's cylinders, No. 1, inserted by means of hand instruments and steel engine instruments. The filling had been made in a German silver matrix, fitting closely around the tooth. The gold had not been annealed, except the last layer upon the grinding surface. The filling and tooth were sawed in two for the purpose of showing the adaptation and solidity of the filling.

No. 2 represented a lower first molar with a large contour filling made in a German silver matrix, and the gold introduced by means of hand instruments and agate engine instruments. The gold used was Wolrab's No. 0 cylinders, which were only annealed in the last layers. The filling appeared to be exceedingly hard, and represented a beautifully smooth polished surface.

No 3 represented a lower second molar with a large contour filling made of Wolrab's gold cylinders No. 0, and weighing six and a half grams. This filling was inserted in a German silver matrix by means of hand and agate engine instruments. This, as well as the foregoing, presented a beautiful appearance, the surface of the filling being hard and nicely polished, without any visible indentations.

No. 4 represented an upper bicuspid with a filled cavity, involving the distal and a portion of the grinding surface of the tooth. The filling had been made in a nickel matrix, and condensed with hand and blood-stone engine instruments. The gold used was No. 1 Wolrab's cylinders, without annealing. This filling showed a beautifully polished surface, upon which, even by the application of a magnifier, no indentations were visible.

No. 5 represented a bicuspid tooth filled with Wolrab's gold cylinders Nos. 1 and 2, inserted in a German silver matrix by means of hand and steel engine instruments. All the gold had been annealed previous to its introduction. The cavity involved a portion of the buccal cusp, as well as mesial and distal portions of the tooth. The filling appeared exceedingly dense, with fine smooth edges.

Preparation No. 6 represented a gold filling made of Wolrab's gold cylinders No. 2, taken out of the tooth and hammered flat, and afterwards, by means of a pair of metal shears, cut into small strips, to prove that gold can be united into a solid mass by the Herbst method, without annealing.

Preparation No. 7 represented a piece of gold sawed off from a large filling made by the Herbst method with Wolrab's gold cylinders. The surface exhibited a homogeneous hard surface, it being untouched by the burnisher or any polishing material.

Preparation No. 8 represented a filling made in the distal cavity of a bicuspid tooth, which had afterwards been cut into thirty-two pieces by means of a pair of metal shears. The filling was made of Wolrab gold, introduced by the Herbst method.

No. 9 represented a very large contour filling, made in a lower molar, and afterwards sawed into three pieces. The grinding surface of the filling had been sawed off first, and the rest of the filling was divided longitudinally; the grinding surface of this filling had been left intact, but the other two portions had been hammered out into pieces measuring three-quarters of an inch in diameter. This was to show that the several layers of the gold, introduced by the Herbst method of filling, were intimately connected with one another. The filling when removed from the tooth represented a weight of eleven grams, and it was made of Wolrab's No. 0 cylinders, without annealing.

Preparation No. 10 represented a mass of gold weighing four grams, condensed by the Herbst method, in a nickle matrix. After the removal of the matrix the gold had been annealed and hammered out on one side, without any apparent check or break in the mass.

Preparation No. 11 represented a large head, measuring about three-quarters of an inch in diameter, which had been condensed into an engraved stone matrix. The gold was made in one layer of 50 cylinders of Wolrab's No. 0, and had been condensed by means of an agate point in the dental engine. The contour of this head was very sharp, and nearly perfect on all its edges.

There was also a preparation which had been made by Dr. L. Foerberg, of Stockholm, which represented the largest contour filling of the whole cabinet. It involved the entire labial and both mesial and distal surfaces, as well as a portion of the cutting edge of an upper central incisor. The filling extended about an eighth of an inch above the edge of the enamel, and the only enamel visible was that on the lingual portion. The surface of this gold, which was condensed by the Herbst rotary motion, was exceedingly hard and dense, and presented a beautifully polished surface.

